# Low Response Rate to Follow-Up Using Telemedicine after Total Knee Replacement during the COVID-19 Pandemic in Italy

**DOI:** 10.3390/jcm13020360

**Published:** 2024-01-09

**Authors:** Eugenio Cammisa, Matteo La Verde, Federico Coliva, Antongiulio Favero, Iacopo Sassoli, Stefano Fratini, Domenico Alesi, Giada Lullini, Stefano Zaffagnini, Giulio Maria Marcheggiani Muccioli

**Affiliations:** 1 Operational Unit of Orthopedics and Traumatology, Imola Hospital Santa Maria della Scaletta, 40026 Imola, Italy; e.cammisa@ausl.imola.bo.it; 2 II Orthopaedic and Traumatology Clinic, IRCCS Istituto Ortopedico Rizzoli—DIBINEM, University of Bologna, 40126 Bologna, Italy; matteo.laverde@ior.it (M.L.V.); federico.coliva@ior.it (F.C.); antongiulio.favero@ior.it (A.F.); iacopo.sassoli@ior.it (I.S.); stefano.fratini@ior.it (S.F.); stefano.zaffangini@unibo.it (S.Z.); 3 IRCCS Istituto delle Scienze Neurologiche di Bologna, UOC Medicina Riabilitativa e Neuroriabilitazione, 40139 Bologna, Italy; giada.lullini@isnb.it

**Keywords:** TKA, knee arthroplasty, telemedicine, COVID

## Abstract

Background: This study aimed to evaluate the survival rate and medium-term outcomes of patients after cemented posterior-stabilized (PS) mobile-bearing (MB) total knee arthroplasty (TKA) using a telemedicine platform during the COVID-19 pandemic in Italy. Methods: A total of 100 consecutive patients (mean age 73.5 ± 13.2 years) who received a cemented PS MB TKA were enrolled. The mean age of patients who did not complete the telemedicine follow-up (58%) was 75.8 ± 9.7 years. A dedicated software that makes it possible to perform video calls, online questionnaires, and acquire X-rays remotely was used. Subjective clinical scores and objective range-of-motion (ROM) measurements were observed at an average follow-up of 54 ± 11.3 months. Results: A total of 42 of 100 enrolled patients (mean age 70.3 ± 8.4 years) completed the telemedicine follow-up. The mean age of patients who did not complete the telemedicine follow-up (58%) was 75.8 ± 9.7 years. Age was found to be a statistically significant difference between the group that completed the telemedicine follow-up and the one that did not (*p* < 0.004). KOOS scores improved from 56.1 ± 11.3 to 77.4 ± 16.2, VAS scores decreased from 7.2 ± 2.1 to 2.8 ± 1.6, KSSf scores increased from 47.2 ± 13.3 to 77.1 ± 21.1, FJS scores improved from 43.4 ± 12.3 to 76.9 ± 22.9, and OKS scores increased from 31.9 ± 8.8 to 40.4 ± 9.9. All the differences were statistically significant (*p* < 0.05). The mean flexion improved from 88° ± 8° to 120° ± 12°. A radiographic evaluation showed a mean pre-operative mechanical axis deviation of 5.3 ± 8.0 degrees in varus, which improved to 0.4 ± 3.4 degrees of valgus post-operation. The survivorship at 5 years was 99%. Conclusions: Subject to small numbers, telemedicine presented as a useful instrument for performing remote monitoring after TKA. The most important factor in telemedicine success remains the patient’s skill, which is usually age-related, as older patients have much more difficulty in approaching a technological tool.

## 1. Introduction

Total knee replacement (TKA) is a common surgical procedure for the management of end-stage knee osteoarthritis, providing significant pain relief and improved function for patients [1]. However, the COVID-19 pandemic has presented unprecedented challenges to healthcare systems worldwide, disrupting routine healthcare services and necessitating innovative approaches to patient care. Long-term follow-up visits for TKA patients have been particularly affected, with limitations on in-person consultations due to infection control measures and patients’ concerns about potential exposure to the virus [2,3].

Regular participation in follow-up visits after total knee arthroplasty is an important aspect of post-TKA patient care. Proper rehabilitation is a crucial part of post-operative recovery, so after each surgery, periodic visits allow the patient’s rehabilitation course to be evaluated and, if needed, modified according to the doctor’s experience and knowledge. At our institution, two years after the TKA surgery, when the rehabilitation period is over and the operation’s clinical results are considered stable, follow-up visits should take place yearly. At every visit, a clinical examination of the patient is performed in which the function and stability of the operated knee are assessed, and, in addition, X-ray images taken in the days close to the visit are evaluated by the orthopedic surgeon to assess if an implant-related complication, such as mobilization of the components, has occurred.

The TKA patient population is often over 60 years of age, and frequently, as these types of surgeries are usually performed in specialized centers, patients must travel long distances from their homes to reach their referral surgical center. Distance, therefore, can become a problem for the patient’s regular follow-up checkups.

In response to these challenges, telemedicine has emerged as a promising solution, enabling remote clinical follow-up without the need for physical examinations [4,5]. Telemedicine offers a means to assess patients’ post-operative progress, monitor outcomes, and provide necessary interventions while minimizing the risk of viral transmission [6]. It involves the use of telecommunication technologies, such as video consultations and remote monitoring devices, to facilitate virtual interactions between healthcare providers and patients [7,8,9].

To the best of our knowledge, there are no recent studies in the literature that have used telematics platforms to specifically analyze outcomes related to primary TKA. The primary aim of this study was to evaluate the survival rate and medium-term clinical outcomes of patients who underwent cemented posterior-stabilized (PS) mobile-bearing (MB) TKA using a telemedicine platform. As a secondary outcome of our study, we analyzed the efficiency of telemedicine as a follow-up instrumentation.

By utilizing telemedicine, we sought to overcome the challenges posed by the COVID-19 pandemic and assess the effectiveness of this approach in facilitating follow-up care for TKA patients.

## 2. Materials and Methods

### 2.1. Study Design

This study utilized a retrospective cohort design to evaluate the survival rate and medium-term clinical outcomes of patients who underwent cemented posterior-stabilized (PS) mobile-bearing (MB) total knee arthroplasty (TKA) using a telemedicine platform. A total of 100 consecutive patients who received the same cemented PS MB Attune TKA (DePuy^®^, Warsaw, IN, USA) between 2015 and 2017 were recruited for the study.

### 2.2. Data Collection

Pre-operative data were collected in person before the surgery with questionnaires routinely administered to all patients during their hospitalization at our institution, thus corresponding to the patient’s state of health the day before the operation. Subjective scores included the Visual Analog Score (VAS) for pain, Knee Society Score Functional Score (KSSf), Oxford Knee Score (OKS), Knee Injury and Osteoarthritis Outcome Score (KOOS), and Forgotten Joint Score (FJS). The pre-operative joint range of motion (ROM) is also derived from the clinical examination carried out on the patient during admission. Furthermore, each patient underwent radiographic investigations before surgery: full weight-bearing radiographs of the lower limbs and a lateral knee radiograph were recorded before surgery.

### 2.3. Use of Telemedicine

Post-operative data were collected through telemedicine visits and involved the collection of subjective scores, radiographic documentation, and reporting of post-operative complications. Firstly, all the patients who underwent TKA with cemented PS MB Attune TKA (DePuy^®^, Warsaw, IN, USA) systems were contacted with a phone call, and the information about their general and specific knee health was recorded to report any implant failure or revision surgery. All the patients who accepted to join the telemedicine follow-up group were enrolled. The patients who refused to undergo the telemedicine follow-up underwent the “standard” follow-up protocol that we apply at our institution with periodic ambulatory evaluations. For long periods, during the COVID pandemic, ambulatory evaluations were not possible because of the contact limitation protocols, and therefore, the non-enrolled group of patients was clinically followed up only when possible. The results presented in our study refer to the 42 patients who were enrolled in the telemedicine follow-up group.

A first telemedicine consultation was performed through a video call: the patient was asked to perform a short walk, visible through the camera of the electronic device, and then lie supine to show the doctor what joint ROM of the knee had been achieved. Once the video call was over, a first report was taken, and the prescription for new X-rays was sent to the patient via e-mail. Each patient underwent full weight-bearing radiographs of the lower limbs and a lateral radiograph of the knee.

**Figure 1:**
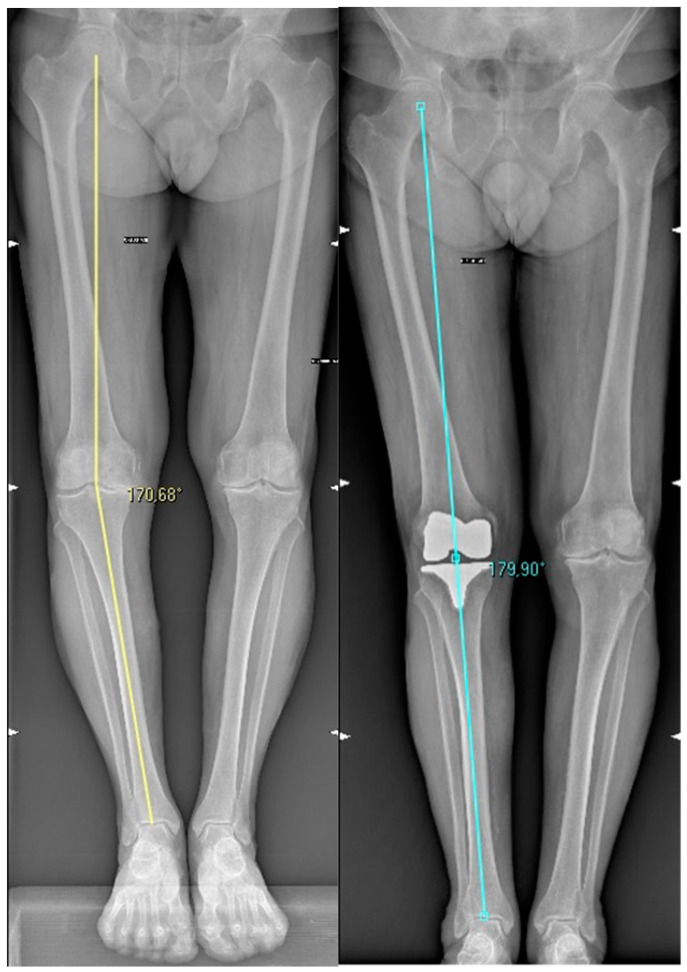
Pre- and post-operative HKA.

The second call was made once the imaging exams had been carried out by the patient, and radiographic documentation was then acquired via a dedicated online software. The telemedicine platform facilitated remote clinical follow-up visits. Patients were able to connect with healthcare professionals through secure video consultations, allowing evaluation of the post-operative progress and the communication of necessary interventions when needed. Before each telephone contact, the privacy consent was explained and displayed in detail, and the call was only initiated once the patient had approved and signed it. The telemedicine platform also allowed the administration of questionnaires to collect subjective scores and provided a tool to review radiographic documentation remotely. A final report was issued after assessing the clinical and radiological data obtained; further investigations or treatments were prescribed if needed. Possible loosening of the prosthetic components was evaluated by comparison with the post-operative X-ray and the most recently collected images, and the hip-knee angle (HKA) was assessed (Figure 1).

**Figure 2:**
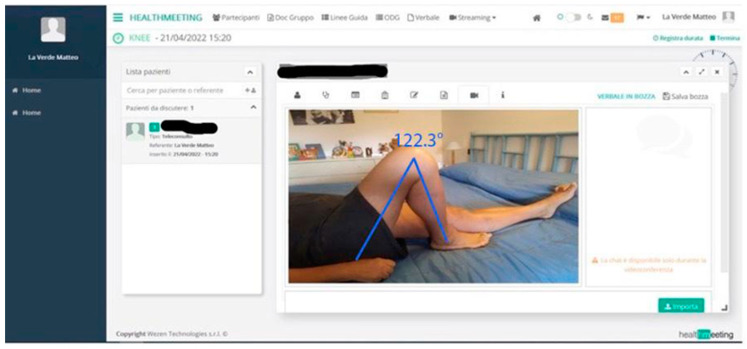
Acquisition of a frame with the telemedicine software during a video call with the patient. The frame is then analyzed to calculate the maximum flexion angle.

Knee ROM was evaluated pre-operatively through an examination conducted in person by the surgeon and post-operatively through the use of the telemedicine platform during the video call, by taking screen-shots of the patient’s image with the knee extended and flexed at its maximum degree as shown in Figure 2 [10].

### 2.4. Statistical Analysis

Descriptive statistics were used to summarize the demographic characteristics of the study population.

Statistical analysis was completed using IBM SPSS Statistics 21.0 (IBM, Armonk, NY, USA), and all results were expressed as mean ± standard deviation (SD). The conformity of the data to the standard distribution was tested by using the Shapiro–Wilk test. Statistical significance was tested using the independent sample *t*-test and one-way analysis of variance, whilst the difference between variables with a non-normal distribution was evaluated using the Mann–Whitney U test. A *p*-value equal to or less than 0.05 was considered significant.

Paired *t*-tests were utilized to compare the pre-operative and post-operative clinical scores. Survival analysis was conducted using Kaplan–Meier estimation, with the survival rate calculated at a specific follow-up duration.

### 2.5. Ethical Considerations

This study was conducted in accordance with the principles outlined in the Declaration of Helsinki and received approval from the relevant institutional ethics committee: PG 0005196-CE AVEC: 230/2021/Oss/IOR. Informed consent was obtained from all participants prior to their inclusion in the study.

## 3. Results

A total of 100 consecutive patients who underwent cemented PS MB Attune TKA between 2015 and 2017 were included in the study. All 100 enrolled patients (mean age at follow-up 73.5 ± 13.2 years) responded to our first follow-up call. A total of 42 patients (mean age 70.3 ± 8.4 years) were able to complete the second visit using the telemedicine platform. The remaining 58 patients (mean age 75.8 ± 9.7 years) refused to undergo the online examination due to technical impediments related to their skills.

The difference in age between the two groups was statistically significant with *p* < 0.004.

Among the 42 patients who completed the telemedicine follow-up, significant improvements in subjective clinical scores were observed at an average follow-up of 54 ± 11.3 months compared to pre-surgery as shown in Table 1.

The pre-operative visual analog scale (VAS) for pain was 7.2 ± 2.1 and the post-operative value was found to be 2.8 ± 1.6 (*p* < 0.0001). The Knee Society Score function (KSSf) improved from a mean pre-operative value of 47.2 ± 13.3 to a post-operative mean value of 77.1 ± 21.1 (*p* < 0.0001). The Oxford Knee Score (OKS) and Knee Injury and Osteoarthritis Outcome Score (KOOS) improved, respectively, from 31.9 ± 8.8 and 56.1 ± 11.3 to 40.4 ± 9.9 and 77.4 ± 16.2 (*p* < 0.0001 for both results). The Forgotten Joint Score (FJS) improved from a pre-operative value of 43.4 ± 12.3 to a post-operative mean value of 76.9 ± 22.9 (*p* < 0.0001). The mean pre-operative Hip Knee Angle (HKA) was 5.3 ± 8° varus and the post-operative mean value obtained was 0.4 ± 3.4° valgus (*p* < 0.0001).

The radiographic evaluation showed a mean pre-operative mechanical axis deviation of 5.3 ± 8.0 degrees in varus, which improved to 0.4 ± 3.4 degrees of valgus post-operation (*p* < 0.0001).

The mean flexion improved from 88° ± 8° before surgery to 120° ± 12° at follow-up.

One patient experienced aseptic loosening of the tibial component and underwent revision surgery, resulting in an overall survivorship at 5 years of 99%. Additionally, four cases of post-operative deep vein thrombosis and one case of pulmonary embolism were reported. The overall complication rate was 6%.

## 4. Discussion

The COVID-19 pandemic has been, for sure, an unexpected global condition that has suddenly changed our daily habits and routines. Even our professional lives have been significantly affected, and contact-limiting measures have changed our usual clinical practice. These global conditions implemented IT systems development, especially telemedicine software, allowing us, clinicians and surgeons, to partially overcome the pandemic-related difficulties.

During the pandemic period, contact-limiting measures have seriously affected medical visits and consultations, especially the ones considered by the patients to be less important, such as the medium- and long-term follow-up ones.

Telemedicine itself can be a powerful system to allow both patients and physicians to overcome not only difficulties related to contact limitations but also other more common problems, for example, distance-related ones. In-person clinical and physical examinations have a non-replaceable value, but the real effectiveness of telemedicine still needs to be evaluated, and further investigations and studies are needed.

### 4.1. Posterior-Stabilized Mobile-Bearing TKA

TKA is one of the most common procedures performed in orthopedic elective surgery, and nowadays, various designs and models are available on the market.

In our study, we tried to evaluate the clinical and radiological midterm results after PS MB TKA using a telemedicine platform to overcome pandemic-related difficulties.

The present study demonstrates that PS MB TKA can achieve favorable midterm clinical outcomes with a high survival rate. The results are consistent with existing literature on TKA outcomes, cemented TKA, and mobile vs. fixed-bearing TKA [11,12]. Many studies have also demonstrated that mobile-bearing designs have specific advantages if compared to other designs. Ueyama H. et al. [11] showed that MB prosthesis suppresses rotational alignment mismatches between the femur and tibia, leading to better patient-related clinical outcomes compared to fixed-bearing TKA, in cases of rotational alignment mismatches.

The findings of this study regarding the clinical outcomes of PS MB TKA using a telemedicine platform align with previous research on total knee arthroplasty (TKA) outcomes. Several studies have reported positive results in terms of pain relief, functional improvement, and patient satisfaction following TKA [13]. The significant improvements observed in subjective scores (such as KOOS, VAS, KSSf, FJS, and OKS) indicate that PS MB TKA is an excellent prosthesis [14,15]. This is confirmed and has already been described in the literature.

Comparing the results of this study to those of existing papers on TKA, particularly studies comparing mobile-bearing (MB) and fixed-bearing (FB) TKA, the PS MB TKA demonstrated comparable outcomes to the PS FB model in terms of subjective and objective clinical results [16]. This supports the notion that the PS MB TKA is a viable alternative with similar functional outcomes. The survival rate of 99% at 5 years further reinforces the effectiveness of the PS MB TKA implant in achieving satisfactory long-term results [16].

Regarding the medium-term outcomes of cemented TKA, the results of this study are consistent with the literature, which indicates favorable clinical outcomes and high implant survival rates for cemented TKA [17]. The low incidence of aseptic loosening observed in this study aligns with the generally accepted success of cemented TKA implants in achieving long-term stability and survival [18].

In terms of complications, post-operative deep vein thrombosis and pulmonary embolism were reported in this study. Although these complications are known risks associated with joint replacement surgery, the incidence observed in this study is within the expected range and can be effectively managed with appropriate prophylactic measures [19].

### 4.2. Telemedicine Follow-Up

The most interesting finding of this study was that 42% of enrolled patients were able to complete the telemedicine follow-up examination. Telemedicine offers advantages in terms of remote follow-up and data collection, but challenges related to patient skills and limitations in the online examination should be acknowledged. The data of our research showed that younger patients were able to complete the telemedicine follow-up with a statistically significant difference (*p* < 0.004) if compared to elderly patients. This is certainly an important factor that needs to be considered if a telemedicine follow-up is offered to our patients.

The integration of telemedicine will offer several advantages in the field of joint replacement surgery. It enables remote clinical follow-ups, reduces the need for in-person visits, and simplifies the administration of questionnaires and reviews of radiographic documentation.

To further optimize the implementation of telemedicine in joint replacement surgery, ongoing research and technological advancements are needed. Studies investigating the validity and reliability of telemedicine assessments, as well as strategies to overcome barriers and improve patient engagement, will contribute to the continued growth and effectiveness of telemedicine in this field.

The integration of telemedicine in the field of joint replacement surgery, including TKA, has been rapidly evolving. Telemedicine offers several advantages, such as facilitating remote clinical follow-ups and reducing the need for in-person visits, particularly during times of crisis such as the COVID-19 pandemic [20]. The logistical simplicity of administering questionnaires and reviewing radiographic documentation through telemedicine platforms improves patient convenience and compliance. Moreover, telemedicine enables healthcare providers to monitor patient progress, address concerns, and provide timely interventions, ultimately enhancing patient care and satisfaction.

Russel T G et al. [21] published in 2004 a paper about the patient and therapist experience with a telemedicine rehabilitation protocol already reporting good results. In the last 20 years, telemedicine systems have strongly improved, and many more devices have been developed.

Agostini M et al. analyzed the use of a telematics platform to perform telerehabilitation [9] and found that certain types of surgery are more suitable for a tele-follow-up pathway than others; for example, orthopedic procedures are more appropriate for a telerehabilitation procedure than patients who have undergone neurological surgery. The use of telemedicine, if aimed at the right target group of people and problems, can lead to excellent results and improve or at least simplify and reduce the costs and time that would be necessary with the conventional methods.

Pang D et al. [22] recently published a paper overviewing the efficacy and the results of telerehabilitation following TKA. Their work included 13 systematic reviews and meta-analyses that showed similar effects of telerehabilitation if compared to conventional rehabilitation protocols regarding the patients’ walking ability and knee extension. Moreover, four papers included in their research analyzed the patient’s post-operative costs, and telerehabilitation was able to reduce medical costs compared to the common protocols. Also, Azma K et al. [23] have compared telerehabilitation results to office-based conventional rehabilitation protocols and obtained comparable clinical results between the two groups with lower time and costs required in the telerehabilitation protocol group.

The efficacy of telerehabilitation protocols has been confirmed and evaluated by many other recent studies [11,24,25,26].

Other possible uses of telemedicine in TKA patients’ care have been evaluated. Lebleu J et al. [27] suggested a possible use of a digital follow-up tool as a system to shorten immediate post-operative hospitalization. In their protocol, patients treated with TKA or Total Hip Arthroplasty (THA) were discharged from the hospital 1 day after surgery. The patients were followed up for 6 weeks with a digital tool, and information about their clinical status, pain, and pictures of the wound and medications were taken and recorded. Their protocol included a pre-operative period at the hospital in which an individualized education session was performed to increase the patient’s skills and abilities with the telemedicine tool. The clinical results of the telemedicine follow-up group were compared to a retrospective cohort of patients who underwent the same surgical procedure and who were followed up with a standard protocol. The complication and readmission rate found was equal between the two groups (2%), and no statistically significant difference was found between the clinical outcomes.

In our experience with telemedicine follow-ups, we found that many patients were not able to complete the visit and exam uploading because of their informatic skills, and usually, they required help from younger relatives to complete the process. Adding an individualized educational session at the moment of hospitalization may be helpful in overcoming these difficulties and allowing the patients to be more confident with the telemedicine instruments. This could also shorten the duration of the telemedicine visits and facilitate the X-ray uploading process.

In 2022, Guida S. et al. [28] published their results with telemedicine follow-ups of TKA and THA. In their paper, they analyzed the main factors correlated to patients’ satisfaction after telemedicine follow-ups. The most significant factors found were related to age and distance. Patients younger than 80 years were mostly satisfied or very satisfied with their telemedicine follow-up, and at the same time, satisfaction was found to be higher in those patients living far away from their referral clinical center. Not surprisingly, the majority of the patients still expressed a preference for in-person visits anyway.

These findings and data suggest that telemedicine is a valuable option and system for the TKA follow-up process, but at the same time, improvements are required. Probably not every patient is suitable for an efficient tele-follow-up, and our study and many others suggest that age is one of the most important parameters that need to be considered in this choice. At the same time, the more telemedicine software and systems become easy to use, the more patients will be encouraged to follow the telemedicine protocols.

However, it is important to acknowledge the drawbacks and limitations of telemedicine in the context of joint replacement surgery.

Technical impediments related to patients’ skills and access to technology can result in low follow-up rates, as observed in this study. Elderly patients may face challenges in adapting to telemedicine platforms, leading to potential selection bias and reduced generalizability of the study findings. Furthermore, the lack of physical examination in telemedicine visits may limit the assessment of certain clinical parameters, such as range of motion and joint stability, which are typically evaluated in an in-person setting.

Regarding the present study, similar pitfalls are present. Firstly, the retrospective design introduces inherent limitations, such as potential selection bias and incomplete data collection. Secondly, the sample size was relatively small, limiting the generalizability of the findings. Additionally, technical impediments related to patients’ skills in using the telemedicine platform resulted in a low follow-up rate, potentially impacting the representativeness of the study population.

## 5. Conclusions

This study highlights the efficacy of utilizing telemedicine for clinical investigation and data collection in the context of TKA follow-ups during the COVID-19 pandemic in Italy. Subject to small numbers, the results of our study suggest telemedicine to be a valuable option for follow-ups in younger patients who are treated with TKA; at the same time, our results confirm the efficacy of PS mobile-bearing cemented TKA as a treatment option for end-stage tricompartmental knee arthrosis.

Acknowledging the limitations and leveraging the advantages of telemedicine, clinicians and researchers can enhance the accessibility and quality of post-operative care, particularly in challenging circumstances such as the COVID-19 pandemic. The most important factor in telemedicine success remains the patient’s skill, which is usually age-related, as older patients have much more difficulty in approaching a technological tool. Further studies are warranted to validate and refine the use of telemedicine in joint replacement surgery.

## Figures and Tables

**Table 1:** Values are presented as mean ± standard deviation (SD).

Variable	Pre-Surgery (Mean ± SD)	Follow-Up (Mean ± SD)	*p*-Value
Visual Analog Score (VAS) for pain	7.2 ± 2.1	2.8 ± 1.6	<0.0001
Knee Society Score Functional Score (KSSf)	47.2 ± 13.3	77.1 ± 21.1	<0.0001
Oxford Knee Score (OKS)	31.9 ± 8.8	40.4 ± 9.9	<0.0001
Knee Injury and Osteoarthritis Outcome Score (KOOS)	56.1 ± 11.3	77.4 ± 16.2	<0.0001
Forgotten Joint Score (FJS)	43.4 ± 12.3	76.9 ± 22.9	<0.0001
Hip-Knee Angle (HKA)	5.3 ± 8° varus	0.4 ± 3.4° valgus	<0.0001
Post-operative complications	-	1 aseptic loosening, 4 deep vein thrombosis, 1 pulmonary embolism	
Flexion	88° ± 8°	120° ± 12°	

This table provides a concise overview of the clinical outcomes and complications observed in the study population. It includes subjective scores such as the Visual Analog Score (VAS) for pain, Knee Society Score Functional Score (KSSf), Oxford Knee Score (OKS), Knee Injury and Osteoarthritis Outcome Score (KOOS), and Forgotten Joint Score (FJS). Additionally, the Hip-Knee Angle (HKA) and post-operative complications are listed. The pre-surgery values are compared to the mean values obtained during the follow-up period.

## Data Availability

The data presented in this study are available in the Section 3 and Table 1.
